# Supplementary figures and images for: Small heat shock proteins determine synapse number and neuronal activity during development
Source: PLoS One. 2020 May 21;15(5):e0233231. doi: 10.1371/journal.pone.0233231 (PMC7241713; doi:10.1371/journal.pone.0233231)

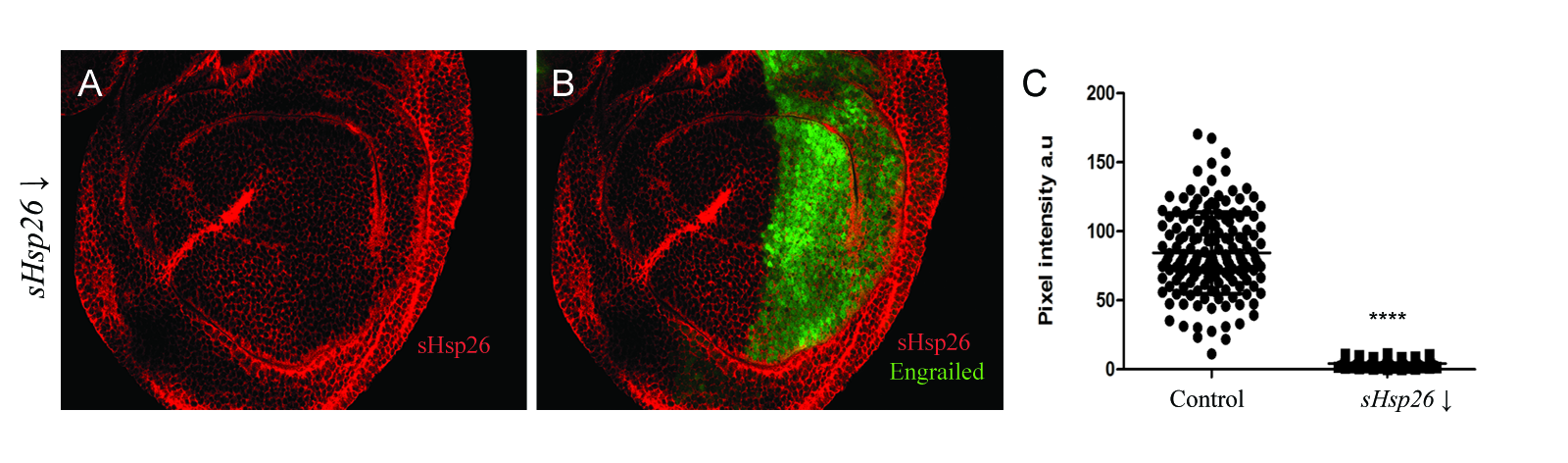

Supplement: S1 Fig — (A-B) To validate if the antibody that we generated against sHsp26 is specific, we knocked down sHsp26 in the posterior compartment of wing imaginal disc (engrailed-Gal4) and visualized the specific domain with the co-expression of GFP. (C) The quantifications of pixel intensity show that anti-sHsp26 recognizes the reduction of sHsp26 expression caused by UAS-sHsp26 RNAi. Unpaired T-test Welch´s correction **** p value<0,0001. Error bars show S.D. (TIF) [file pone.0233231.s001.tif]
