# Supplementary material for: Small heat shock proteins determine synapse number and neuronal activity during development
Source: PLoS One. 2020 May 21;15(5):e0233231. doi: 10.1371/journal.pone.0233231 (PMC7241713; doi:10.1371/journal.pone.0233231)

ORIGINAL IMAGES FOR BLOTS AND GELS

Original file Figure 2. Original file Fig 2G Immunoprecipitation HSP23 and HSP26. Figure 2G in the article is based on 1 to 4 lanes from the original file. HSP23 is marked in red colour and HSP26 in green.

Supplementary Figure 3. Original file Fig 3D Immunoprecipitation HSP23 and HSP26 in *pkm* RNAi context. Figure 3D in the article is based on 1 to 10 lanes from the original file respect to HSP23 signal. HSP23 is marked in red.

Supplementary Figure 4. Original file Fig 4B Western blot HSP23 and HSp26 in *pkm* RNAi context. Figure 4B in the article is based on 2 and 3 lanes from the original file respect to HSP23 and HSP26 signal. HSP23 is marked in red colour and HSP26 in green.

Same gel with longer exposure time "Original file Figure 4 exposition 2"

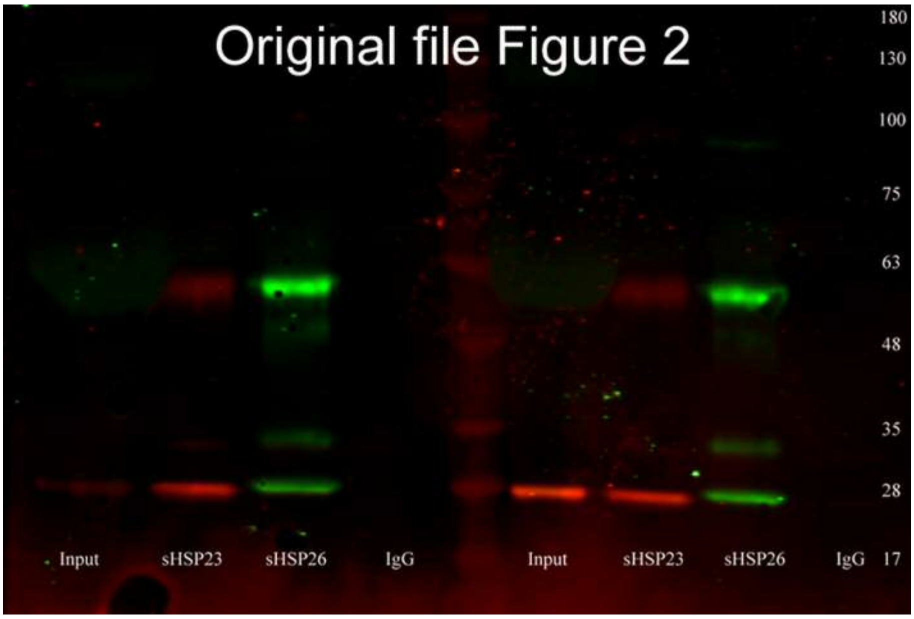

Original file Figure 2
180
130
100
75
63
48
35
28
17
Input
sHSP23
sHSP26
IgG
Input
sHSP23
sHSP26
IgG

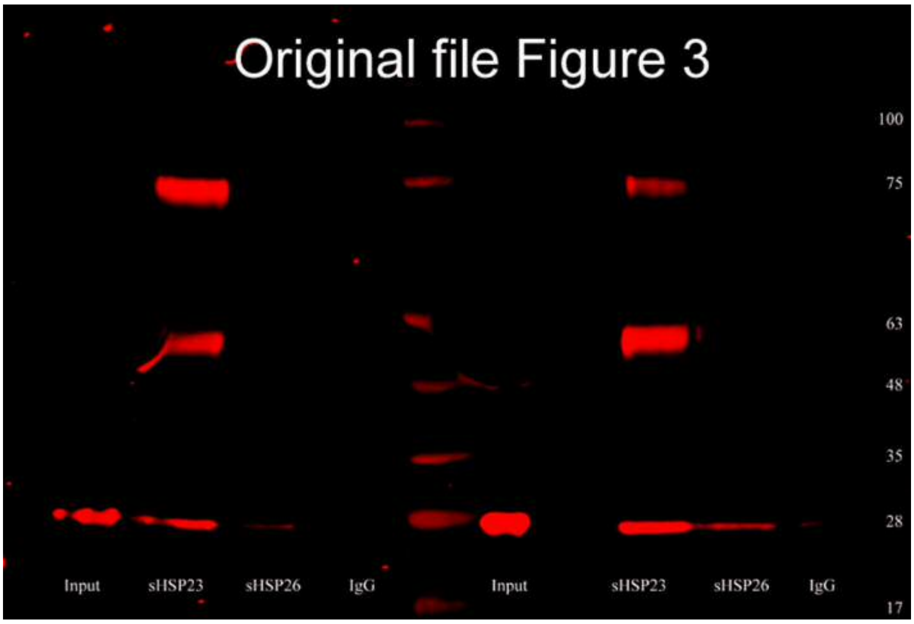
Original file Figure 3
100
75
63
48
35
28
17
Input
sHSP23
sHSP26
IgG
Input
sHSP23
sHSP26
IgG

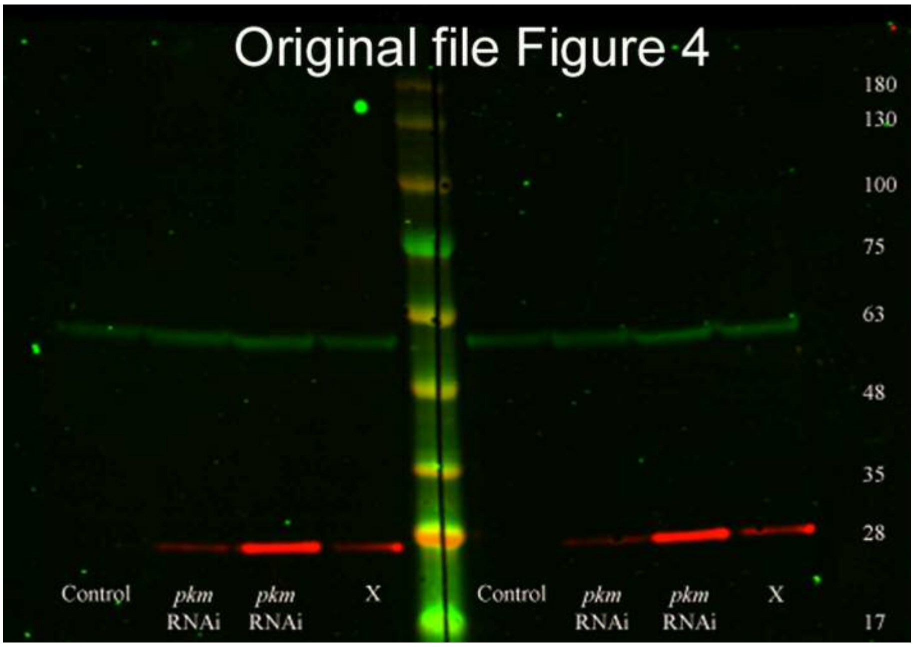
Original file Figure 4
180
130
100
75
63
48
35
28
17
Control
pkm RNAi
pkm RNAi
X
Control
pkm RNAi
pkm RNAi
X

Original File Figure 4 exposition 2

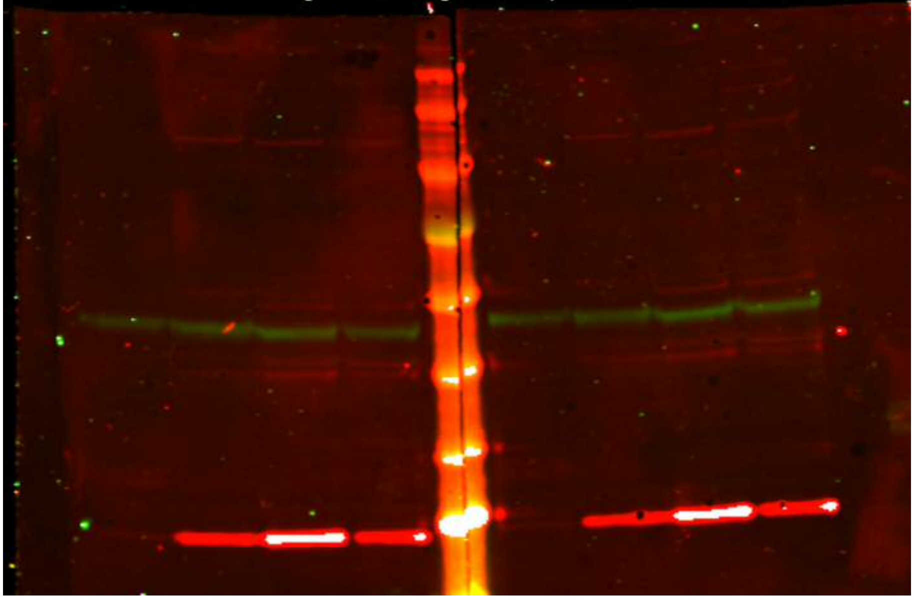

Supplement: S1 Raw images — (PDF) [file pone.0233231.s002.pdf]
